# Ledum in Cutaneous Affections

**Published:** 1882-06

**Authors:** L. B. Wells

**Affiliations:** Utica, N. Y.


### CLINICAL BUREAU.

### LEDUM IN CUTANEOUS AFFECTIONS.

L. B. WELLS, M. D., UTICA, N. Y.

Failure of physicians to keep a record of cures following the administration of remedies when well indicated, is a reason why our materia medica is no better supplied with clinical curative marks. How often we find one or more important symptoms of a case and the symptoms of the corresponding remedy in plain type more plainly indicated and consequently more curative. If physicians would keep a record of these well-marked cures an important addition would be made to our armamentarium in confirmation of provings. Ledum in my hands has proved a complete curative in suggillations which often attend contusions and remain frequently for weeks, annoying to the patient, especially on exposed parts.

In 1849, Mr. O. W., aged 50, when passing over a stream, a dark night, accidentally stepping in a hole in the bridge, caused a severe contusion of almost the entire leg, from the knee to the ankle. The pain was severe and inflammation followed, which was relieved by Arnica, and Aconite afterwards. Some three weeks afterwards he called at my office, the injured parts discolored. He said to me, "If there is anything in homœopathic medicine that could remove the discoloration he wanted it."

I searched Jahr's *Manual*, and the nearest symptom I could find was under Ledum, "Bluish spots like petechiæ over the whole body." This was given in the 6th potency and in four days the discoloration was entirely removed.

Mrs. D., aged 74, while standing on a chair, fell and her nose came in contact with the edge of the chair. Arnica relieved the pain and soreness and the effects of the shock, but the extravasated blood caused a discoloration of both cheeks. A kind neighbor informed her that the natural color would not return in a person of her age.

Ledum was administered and in a very few days all traces of the discoloration vanished.

I select these two cases from many of the kind and I can truly say that there has not been a single failure.

